# How Do Filamentous Pathogens Deliver Effector Proteins into Plant Cells?

**DOI:** 10.1371/journal.pbio.1001801

**Published:** 2014-02-25

**Authors:** Benjamin Petre, Sophien Kamoun

**Affiliations:** 1 The Sainsbury Laboratory, Norwich Research Park, Norwich, United Kingdom; 2 INRA, Interactions Arbres/Microorganismes, UMR 1136, Champenoux, France; Virginia Tech, United States of America

## Abstract

Fungal and oomycete plant parasites are among the most devastating pathogens of food crops. These microbes secrete effector proteins inside plant cells to manipulate host processes and facilitate colonization. How these effectors reach the host cytoplasm remains an unclear and debated area of plant research. In this article, we examine recent conflicting findings that have generated discussion in the field. We also highlight promising approaches based on studies of both parasite and host during infection. Ultimately, this knowledge may inform future broad spectrum strategies for protecting crops from such pathogens.

## Abstract 2

How potentially devastating fungal effector proteins reach the host cytoplasm is an unclear and debated area of plant research; this Unsolved Mystery discusses the various current opinions and proposes an urgent need for novel experimental approaches.

## Introduction

**Figure 1:**
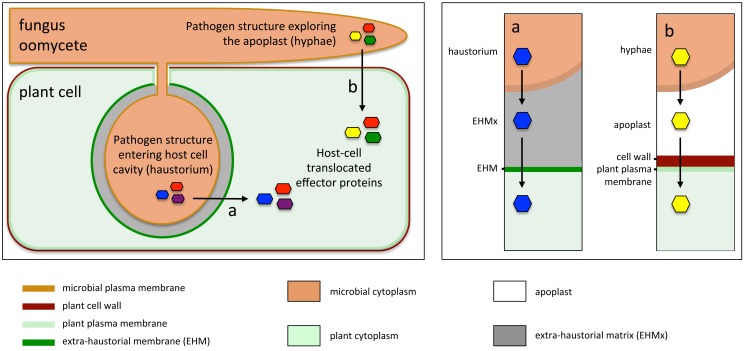
Fungal and oomycete structures for effector secretion. Left panel. Oomycete and fungal plant parasites differentiate infection structures such as extracellular hyphae, as well as invasive hyphae and haustoria that penetrate the host cell cavity and invaginate the plasma membrane. Haustoria (a) and hyphae (b) secrete effectors that are translocated into host cell cytoplasm by unknown mechanisms. Right panel. Effectors secreted from haustoria (a) and hyphae (b) cross different biological interfaces (extra-haustorial matrix [EHMx]/extra-haustorial membrane [EHM] for effectors secreted from haustoria, and apoplast/plant cell wall/plant plasma membrane for effectors secreted from hyphae).

Fungi and oomycetes are eukaryotic filamentous microbes, some of which are devastating plant pathogens that affect important food crops. For instance the oomycete potato blight pathogen *Phytophthora infestans* triggered the Irish famine during the 19th century and remains the most important threat to potato production, whereas fungi such as the ascomycete rice blast pathogen *Magnaporthe oryzae* and the basidiomycete wheat stem rust pathogen *Puccinia graminis* f. sp. *tritici* continuously threaten global food security [1],[2]. During infection, these parasites engage in complete or partial biotrophic interactions, meaning that they develop feeding relationships with the living cells of their hosts by intimately associating with plant tissues. These microbes differentiate specialized parasitic structures within infected tissues, such as hyphae, which explore the extracellular space (apoplast), or invasive hyphae and haustoria, which penetrate host cell cavities and invaginate the host's plasma membrane (Figure 1) [3],[4]. Historically, hyphae and haustoria have been described as feeding structures that serve the nutrition of the parasites. But more recently these structures have emerged as sites of secretion and translocation into host cells of a class of pathogen virulence proteins known as effectors (Figure 1) [5],[6].

Effectors manipulate plant processes to the advantage of the parasite, promoting host infection and colonization, yet they may also activate plant immune receptors on resistant host genotypes [7]. During the past decade, it has become apparent that numerous fungal and oomycete effectors operate inside the host cell cytoplasm [8]–[11], extending to these pathogens a concept first put forward for plant pathogenic bacteria [12]. Nevertheless, the mechanisms by which effector proteins traffic to the plant cell cytoplasm remain poorly understood in contrast to the well-studied bacterial secretion systems. Solving the enigma of how filamentous pathogens deliver their effectors to the inside of plant cells is a fundamental question in plant pathology. Moreover, the prevention of effector secretion or internalization into host cells is likely to interfere with parasitic growth, thus representing a potential crop protection strategy for use in agriculture. Also, effectors target different host subcellular compartments and mediate a variety of biochemical modifications, thus representing valuable molecular tools for fundamental and applied plant biology studies [7],[13].

**Figure 2:**
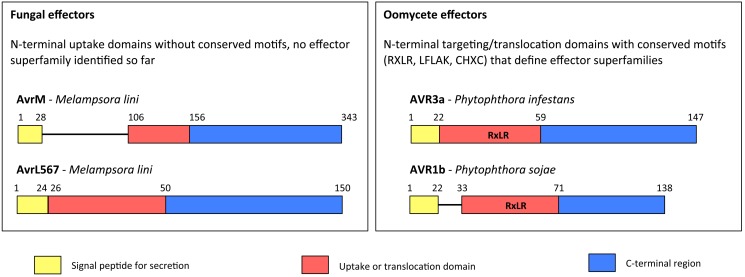
N-terminal effector domains proposed to mediate host-cell entry. Effectors from fungal (left) and oomycete (right) pathogens. Divergent oomycete and fungal effectors carry a general secretion signal peptide followed by non-conserved N-terminal regions called “uptake” or “targeting/translocation” domains that have been proposed to mediate host-cell entry. In oomycetes, small conserved amino acids motifs (e.g., RXLR, CHXC, or LFLAK) have been identified within these regions, which help to define effector families with many members.

Filamentous pathogen effector proteins that translocate into plant cells are highly diverse in sequence and structure and have most likely evolved a variety of mechanisms to traffic to the host cytoplasm. However, a common theme is that host-targeting relies on N-terminal translocation domains that are located after a general secretory signal peptide (Figure 2). In the oomycetes, host-targeting domains contain overrepresented motifs, such as the RXLR, LFLAK, and CHXC amino acid sequences, which define many predicted effector repertoires in different species [14]. In one early study, Whisson and colleagues (2007) showed that the N-terminus of the AVR3a effector from *P. infestans* is required for translocation into potato cells, a finding that supported the view that the RXLR domain functions as a leader sequence that mediates host cell targeting [5].

Identification of motifs involved in cell entry is not as advanced for fungal effectors as it is for oomycetes. Large families of candidate effectors have been identified from fungal genomes, largely on the basis of predicted N-terminal signal peptides, small size, and lack of similarity to other proteins [15],[16]. Additionally, sequences that mediate host-cell translocation have been detected within host-specific toxins of necrotrophic fungi. One well-studied example is the C-terminal RGD motif of ToxA from *Pyrenophora tritici-repentis*, which is required for entry into host plant cells [17]. Also, domains in the N-termini of the flax rust fungus *Melampsora lini* effectors AvrM and AvrL567 mediate uptake into plant cells, although whether these sequences determine entry into plant cells or other processes, such as escape from plant endosomes following endocytosis, is still unclear (see below) [6],[18]. However, a consensus cell entry motif for fungal proteins, analogous to the common RXLR in oomycetes, has not been defined.

## Current Models and Controversies

A number of studies aimed at elucidating the function of N-terminal host-targeting domains of filamentous plant pathogen effectors have been published and are summarized in Table 1. Monitoring effector trafficking from the parasite to the host cell is technically challenging. Indeed, many filamentous plant pathogens are not amenable to genetic manipulation, and the direct visualization of effector proteins during infection has proven to be elusive. In addition, effectors traffic across dynamic interfaces, such as haustoria, that can only form inside host tissue. As a consequence, the results and models generated to date are mostly based on proxy experiments conducted independently of the pathogen. They essentially tackle the question of “how effectors cross the host plasma membrane” (summarized in [19]), leading to a model that involves “autonomous” or “pathogen-independent” host cell entry [6],[20]–[22]. Kale and colleagues (2010) also proposed a mechanistic model for this phenomenon. The RXLR motif in oomycetes or degenerate RXLR-like motifs in fungi define cell entry domains and bind extracellular phosphatidylinositol-3-phosphate (PI3P) to mediate effector endocytosis into host cells [22],[23]. However, the experimental findings that underpin this and related models have proven controversial with several studies alternatively supporting or challenging the reproducibility of the assays and the robustness of the conclusions (see Table 1 for details).

**Table 1:** List of conflicting studies on filamentous pathogen effector translocation inside plant cells.

Articles	Main Conclusions	Effectors Examined	Assays Used			Findings Reported^a^		
			Cell Re-entry	Uptake Assay	Phospholipid Binding	Functional RXLR-Like Motifs in Fungal Effectors	Pathogen-Independent Cell entry	RXLR and RXLR-Like Motifs Bind Phospholipids
Catanzariti et al., 2006 [20]	Fungal effectors AvrM and AvrP4 enter flax cells autonomously.	AvrM, AvrP4 (F)	AI (HR)				Yes	
Bos et al., 2006 [38] b	Cell re-entry assays are inconclusive.	Avr3a (Oo)	AI (HR)				Inconclusive	
Dou et al., 2008 [21]	Oomycete effector Avr1b enters soybean cells autonomously; RXLR motif mediates cell entry.	Avr1b (Oo)	PB (HR, FP)	PR (FP)			Yes	
Oh et al., 2009 [28]	Cell re-entry assays are inconclusive.	Avr3a, Avr1b, Avrblb2 (Oo)	AI (HR)				Inconclusive	
Rafiqi et al., 2010 [6]	Fungal effectors AvrM and AvrL567 enter flax and tobacco cells autonomously; divergent N-terminal domains mediate cell entry.	AvrM, AvrL567 (F)	AI (HR, FP)			Inconclusive	Yes	
Kale et al., 2010 [22]	Several oomycete and fungal effectors enter plant and animal cells autonomously via phospholipid-binding mediated endocytosis; oomycete RXLR and fungal RXLR-like motifs mediate binding of phospholipids and cell entry.	Avr1b, Avh5, Avh331 (Oo) AvrM, AvrL567, AvrLm6, Avr2, Avr-Pita (F)	PB (HR)	PR (FP), PL (HR), AC (FP)	DB, LB	Yes	Yes	Yes
Gan et al., 2010 [39] b ^,^ c	C-terminal domain, not the N-terminal uptake domain, of the fungal effector AvrM bind phospholipids; fungal effector AvrL567 does not bind phospholipids.	AvrM, AvrL567 (F)			DB	Inconclusive		No
Yaeno et al., 2011 [8]	C-terminal domain, not the RXLR domain, of oomycete RXLR effectors binds phospholipids; phospholipid binding occurs inside the host cell and stabilizes the effector.	Avr3a, Avr1b, Avr3a4 (Oo)			DB			No
Plett et al., 2011 [23]	Fungal effector MiSSP7 enters poplar cells autonomously via phospholipid-mediated endocytosis; an RXLR-like motif mediates phospholipid binding and cell entry.	MiSSP7 (F)		PR (FP)	DB, LB	Yes	Yes	Yes
Gu et al., 2011 [40] b	Fungal effector candidate Ps87 enters soybean cells autonomously; an RXLR-like motif mediates cell entry.	Avr1b (Oo) Ps87 (F)	PB (HR)	PR (FP)		Yes	Yes	
Bhattacharjee et al., 2012 [31]	The RXLR domain of the oomycete effector NUK10 binds phospholipids.	NUK10 (Oo)			SPR			Yes
Wawra et al., 2012 [32]	The C-terminal domain, not the RXLR domain, of the oomycete effector Avr3a binds phospholipids; denatured Avr3a protein binds phospholipids.	Avr3a (Oo)			DB, ITC			No
Ribot et al., 2013 [41] b	Fungal effector Avr1-CO39 enters rice cells autonomously.	Avr1-CO39 (F)	PEG (FP)				Yes	
Sun et al., 2013 [26]	Both C-terminal residues and the N-terminal RXLR motif of the oomycete effector Avh5 mediate phospholipid-binding and promote autonomous entry into human and soybean cells; principal binding site is in the C-terminus with the RXLR motif playing a minor role.	Avh5 (Oo)		PR, AC (FP)	DB, LB, NMR, SPR		Yes	Yes
Yaeno and Shirasu, 2013 [42] b	The oomycete RXLR effectors Avr3a4, Avr3a11 and ATR1 do not bind phospholipids.	Avr3a4, Avr3a11, ATR1 (Oo)			DB			No
Wawra et al., 2013 [29]	Protein uptake assays fail to demonstrate specific and autonomous RXLR-dependent cell entry of oomycete effectors Avr3a and Avr1b.	Avr3a, Avr1b (Oo)		PR, PL, AC (FP)			Inconclusive	
Tyler et al., 2013 [30]	Oomycete effector Avr1b enters soybean and wheat cells specifically and autonomously; the RXLR motif mediates cell entry on the basis of a quantitative difference with the negative controls.	Avr1b (Oo)		PR, PL (FP)			Yes	
Na et al., 2013 [43] b	The C-terminal domain and not the RXLR domain of the oomycete effector Avr1d binds phospholipids; cell re-entry assays with Avr1d are inconclusive.	Avr1d/Avh6 (Oo)	PB (HR)		LB		Inconclusive	No
Ve et al., 2013 [18]	Positively charged residues of the fungal effector AvrM mediate phospholipid-binding but these residues are not required for cell internalization; a hydrophobic patch in the N-terminus is required for plant cell entry.	AvrM (F)	AI (HR, FP)		DB	No	Yes	No

a Yes, results support finding; No, results do not support finding.

b References not cited in the main text.

c Article addendum.

AC, animal cells; AI, agroinfiltration; DB, dot blot; F., fungal; FP, fluorescent protein; HR, hypersensitive response; ITC, isothermal titration calorimetry; LB, liposome binding; NMR, nuclear magnetic resonance; Oo, oomycete; PB, particle bombardment; PEG, polyethylene glycol; PL, plant leaves; PR, plant roots; SPR, surface plasma resonance.

First, the occurrence of RXLR-like motifs in fungal effectors [22],[23] that are functionally and structurally related to oomycete RXLR motifs is questionable [18]. The RXLR consensus, associated sequence motifs, and their position near N-termini helped to define the RXLR effector superfamily, which includes hundreds of divergent members in the *Phytophthora* species, most of which (87%) carry the RXLR sequence [14],[24]. Although variants have been detected, notably QLLR and GKLR in some downy mildew pathogens, the RXLR motif is highly conserved in *Phytophthora* effectors even though these proteins are rapidly evolving and can display high levels of amino acid polymorphism [14],[24],[25]. This indicates that the RXLR motif is mostly under purifying selection, meaning that variants that have arisen have been mostly eliminated by natural selection. Nonetheless, Kale and colleagues (2010) used extensive mutagenesis studies of this sequence combined with cell re-entry and uptake assays (see next paragraph) to show that the motif is highly plastic and that some fungal effectors carry N-terminal RXLR-like motifs, which are highly degenerate as [RHK]X[LMIFYW] [22]. By using similar assays, some authors reported the existence of functional RXLR-like motifs in various fungal effectors, whereas others did not (Table 1). Interestingly, structural investigations of the oomycete effectors Avr3a4 and Avh5 revealed that RXLR domains are intrinsically disordered [8],[26]. In contrast, RXLR-like motifs of the fungal effectors AvrL567 and AvrM are embedded in well-defined structures [18],[27]. Hence, based on the few structures currently available, amino acids similarities within the effector primary sequences are not matched by their structural properties.

Second, the two main assays used to demonstrate pathogen-independent effector entry into host cells are under debate. One such method, the “cell re-entry assay,” is based on the heterologous expression of a full-length effector protein, including its secretion signal peptide, in a plant cell. The expressed effector, or effector-fluorescent protein fusion, is secreted into the extracellular space (apoplast), and its re-entry into the plant cell is tracked [20]. This method has been used to report autonomous cell entry of several fungal and oomycete effectors and to identify the uptake domains required for entry [6],[21],[22]. Nevertheless, this assay cannot unambiguously demonstrate that effectors are indeed secreted into the apoplast prior to cell re-internalisation [28] and it is therefore not possible to exclude that effectors escape the secretory pathway and end up inside the host cytoplasm without crossing the plasma membrane. This limitation of the cell re-entry assay prompted some authors to complement their experiments with a second assay—the “uptake assay”—in which purified recombinant effectors fused to a fluorescent tag are applied to plant tissue, often roots, and their entry followed by microscopy [21]–[23]. Recently, the robustness and specificity of this method have been debated (Table 1) [29],[30]. Wawra and collaborators (2013) proposed that the process of protein internalization by root cells is non-specific and thus could not inform cell entry mechanisms [29]. Their point was supported by the observation that fluorescent proteins alone are taken up by plant cells at a rate comparable to effector-fluorescent protein fusions. In response, Tyler and colleagues (2013) state that quantitative differences could still be observed, and reported increased entry into cells when fluorescent proteins are fused with effectors or effector uptake domains [30].

Finally, there have been conflicting reports as to whether oomycete RXLR domains can bind phospholipids to mediate cell entry (Table 1). Bhattacharjee and colleagues (2012) confirmed that the RXLR domain of *P. infestans* effector NUK10 binds PI3P but proposed that this binding takes place inside the pathogen [31]. Sun and colleagues (2013) further investigated the *P. sojae* effector Avh5 revealing stronger PI3P binding in the C-terminal domain relative to the RXLR domain, but implicating both regions in cell entry [26]. Other studies showed that amino acids residues in the C-terminal half of some oomycete RXLR effectors, rather than in the N-terminus, bind phospholipids and may have a function unrelated to cell entry (Table 1) [8]. Consistent with this idea, some have proposed that phospholipid binding stabilises effectors [26], possibly inside host cells, rather than onto the external surface of the host plasma membrane [8]. Wawra and collaborators (2012) also showed that phospholipid binding of the RXLR effector Avr3a can occur with denatured proteins, and thus questioning the physiological relevance of phospholipid binding [32].

In conclusion, many aspects of the mechanisms by which fungal and oomycete effectors enter into plant cells remain unresolved. There is therefore an urgent need to complement evidence from proxy assays with novel experimental approaches to shed new light on this process.

## Towards a Solution: Integrated Pathogen-Host Studies

**Figure 3:**
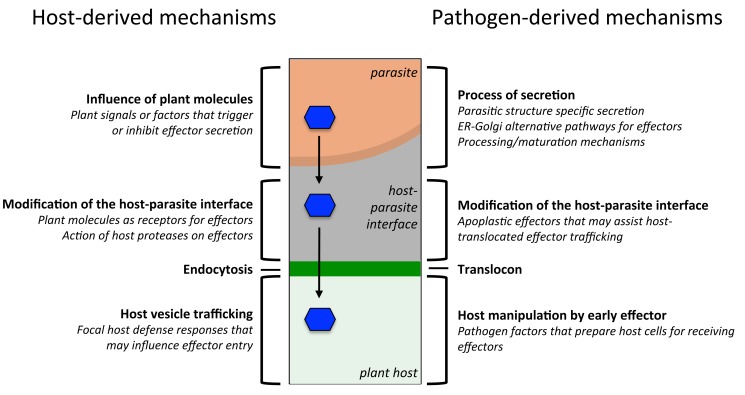
Integrated process of effector translocation. Effectors (blue) follow secretion routes (arrows) within a pathogen (orange), are secreted into host-parasite interfaces (grey), cross a membrane surrounding the host cell (green), and finally enter the host cell cytoplasm. Each translocation step is likely to be influenced by host- and parasite-derived mechanisms that need to be considered when studying effector trafficking.

Our basic understanding of effector trafficking has been hampered by our inability to follow effector secretion and translocation during infection. During translocation, effectors cross several biological interfaces that can be modified during the interaction, as well as new infection-specific compartments (Figure 3) [33]. For instance, haustoria are enveloped by a newly formed membrane called the extrahaustorial membrane (EHM), which differs in protein composition to the plant plasma membrane [34]. These infection-specific biological interfaces are probably mediated by both parasite- and plant-derived factors that need to be taken into account, as they could well influence, if not mediate, effector translocation.

The major challenge for the community is methodological. We therefore need to develop genetic, biochemical, and cell biological methods to manipulate, tag, detect, and observe effectors during infection. A growing number of oomycete and fungal plant pathogens are now genetically transformable, thus enabling more pathogen-centered studies. Examples of the value of pathogen-focused studies come from the interaction between *M. oryzae* and the host plant rice [33] or *Ustilago maydis* and maize [10]. These pathogens produce invasive hyphae that invaginate the host cell plasma membrane. The use of *M. oryzae* strains that express fluorescently tagged effectors combined with live-cell imaging has revealed that a highly localized structure, called the biotrophic interfacial complex (BIC), accumulates effectors secreted from the invasive hyphae prior to translocation into the host cell [11],[35]. Such experimental systems should allow further insight into effector trafficking by, for example, addressing the contribution of specific residues within effectors, the influence of infection conditions on effector translocation, and the degree to which plant-derived molecules affect translocation (Figure 3).

The presence of predicted signal peptides in effector proteins has led to the assumption that effectors follow the typical eukaryotic endoplasmic reticulum (ER)/Golgi secretory pathway. As a consequence, the secretion routes followed by effectors inside the pathogen, prior to their secretion and translocation into host cells, have been poorly studied but could turn out to be important as in the case of apicomplexan parasites [31]. For instance, Yi and colleagues (2009) reported that the ER-resident chaperone LHS1 of *M. oryzae* interferes with effector accumulation at the BIC, and possibly effector secretion [36]. Interestingly, a recent paper combined cell biology with pharmacological approaches to identify two distinct effector secretion pathways in *M. oryzae* [37]. Whereas apoplastic effectors follow the conventional ER/Golgi secretory pathway, host-translocated effectors appear to follow an alternative secretion route. The extent to which effectors from other pathogens are sorted into distinct secretory pathways remains unknown.

Biochemical approaches need to be explored too. For instance, immunoprecipitation of tagged effectors during the course of infection could reveal the formation of effector-associated protein complexes during the different steps of secretion and translocation. Mass spectrometry associated with biochemical cell fractionation should also enable high throughput subcellular localisation of proteins. Such methods could be applied to colonized tissues, and would assign effectors to different plant subcellular compartments, thus providing evidence of secretion and other valuable information for further characterisation [10].

Finally, although the use of proxy assays alone is unlikely to reveal the full process of effector trafficking, they remain the only alternative in several pathogen systems, and could still provide valuable clues. Some of the established methods, such as the plant cell re-entry assays, need to be better understood. For instance stable transgenic plants expressing fluorescently tagged effector proteins driven by cell-specific promoters should be assayed. The precise fate of heterologously expressed effector proteins also needs to be determined using cell biological and biochemical methods, and the use of multiple tagged proteins tested. Moreover, reagents should be shared between labs and there should be less reliance on transient expression assays.

In conclusion, the targeting of pathogen effectors to the cytoplasm of their plant hosts is a complex process that involves numerous steps (Figure 3). Studies to date have provided some valuable information on effector trafficking in many systems, but new methods are needed to uncover a more comprehensive picture of this process—ideally integrated experimental systems that will allow the detection and visualization of effectors as they traffic from the parasite to the host cell.
